# Genome-Wide Association Analysis of Autoantibody Positivity in Type 1 Diabetes Cases

**DOI:** 10.1371/journal.pgen.1002216

**Published:** 2011-08-04

**Authors:** Vincent Plagnol, Joanna M. M. Howson, Deborah J. Smyth, Neil Walker, Jason P. Hafler, Chris Wallace, Helen Stevens, Laura Jackson, Matthew J. Simmonds, Type 1 Diabetes Genetics Consortium, Polly J. Bingley, Stephen C. Gough, John A. Todd

**Affiliations:** 1 Juvenile Diabetes Research Foundation/Wellcome Trust Diabetes and Inflammation Laboratory, Department of Medical Genetics, Cambridge Institute for Medical Research, National Institute for Health Research Biomedical Research Centre, University of Cambridge, Cambridge, United Kingdom; 2 University Hospitals Birmingham, Birmingham, United Kingdom; 3 The Oxford Centre for Diabetes, Endocrinology, and Metabolism, Churchill Hospital, Headington, Oxford, United Kingdom; 4 School of Clinical Sciences, University of Bristol, Bristol, United Kingdom; University of California San Diego and The Scripps Research Institute, United States of America

## Abstract

The genetic basis of autoantibody production is largely unknown outside of associations located in the major histocompatibility complex (MHC) human leukocyte antigen (HLA) region. The aim of this study is the discovery of new genetic associations with autoantibody positivity using genome-wide association scan single nucleotide polymorphism (SNP) data in type 1 diabetes (T1D) patients with autoantibody measurements. We measured two anti-islet autoantibodies, glutamate decarboxylase (GADA, n = 2,506), insulinoma-associated antigen 2 (IA-2A, n = 2,498), antibodies to the autoimmune thyroid (Graves') disease (AITD) autoantigen thyroid peroxidase (TPOA, n = 8,300), and antibodies against gastric parietal cells (PCA, n = 4,328) that are associated with autoimmune gastritis. Two loci passed a stringent genome-wide significance level (*p*<10^−10^): 1q23/*FCRL3* with IA-2A and 9q34/*ABO* with PCA. Eleven of 52 non-MHC T1D loci showed evidence of association with at least one autoantibody at a false discovery rate of 16%: 16p11/*IL27*-IA-2A, 2q24/*IFIH1*-IA-2A and PCA, 2q32/*STAT4*-TPOA, 10p15/*IL2RA*-GADA, 6q15/*BACH2*-TPOA, 21q22/*UBASH3A*-TPOA, 1p13/*PTPN22*-TPOA, 2q33/*CTLA4*-TPOA, 4q27/*IL2*/TPOA, 15q14/*RASGRP1*/TPOA, and 12q24/*SH2B3*-GADA and TPOA. Analysis of the TPOA-associated loci in 2,477 cases with Graves' disease identified two new AITD loci (*BACH2* and *UBASH3A*).

## Author Summary

Autoantibodies are important markers for autoimmune diseases such as type 1 diabetes and Graves' disease. However, little is known about the genetic factors that control their production. To improve our understanding of this genetic basis, we measured four autoantibodies in a collection of up to 8,300 type 1 diabetes cases plasma samples. We combined these measurements with genome-wide genotype data to conduct four independent genome-wide association studies. Two loci showed unequivocal evidence of autoantibody association: the *FCRL3* locus and the *ABO* blood group locus. Variants in the *FCRL3* gene have been previously associated with autoimmune diseases, but such associations have not been reported for *ABO* blood group genotypes. In addition, we found extensive overlap between type 1 diabetes and autoantibody loci, and these findings provide new information about the role of these risk variants. Lastly, we hypothesized that loci associated with thyroid autoantibodies are strong candidates for association with thyroid autoimmune disorders. We confirmed this hypothesis by genotyping these variants in an independent cohort of Graves' disease cases, and we found evidence for two new Graves' disease loci.

## Introduction

The presence of circulating antibodies to the body's own antigens, namely autoantibodies, is the major hallmark of autoimmunity, which can progress to the diagnosis of a variety of autoimmune diseases. Autoantibodies directed to antigens in the pancreatic islets, for example, glutamate decarboxylase (GADA) and islet antigen-2 (IA-2A), are characteristic of type 1 diabetes (T1D). The dynamics of T1D-associated autoantibodies in T1D patients are complex. They are detected prior to clinical diagnosis and often persist several years after diagnosis [1], but they can also disappear prior to T1D diagnosis [2], and, in general, decline from the time of diagnosis onwards. Antibodies are produced by B lymphocytes. The success of B cell depletion therapies in slowing beta-cell destruction in the mouse animal model [3] and more recently the positive effects of such therapies also reported in a clinical trial [4], demonstrate that B cells play a role in T1D pathogenesis. However, it is also generally accepted that anti-islet antibodies are not pathogenic themselves [5], in contrast, for example, to autoantibodies in systemic erythematosus lupus (SLE) [6]. The report of a T1D patient with a severe hereditary B cell deficiency [7], and the fact that in animal models of T1D the disease is transferable to healthy recipients by T cells but not by serum [8], are consistent with this view.

B cell maturation to autoantibody secreting state requires CD4 T helper cells to recognize human leukocyte antigen (HLA) class II molecules bound peptides on the surface of B cells and on other antigen-presenting cells [9]. Concordantly, candidate gene association studies have provided evidence for association of autoantibodies with HLA class II alleles [10], [11]. Outside of these HLA associations, relatively little is known about the genes associated with autoantibody production. However, we can hypothesize that there should be some overlap in the genes and their alleles that increase the risk of T1D with those that show association with autoantibody positivity. If autoantibody positivity *per se* is not a primary causal factor we should also observe T1D risk alleles that do not show evidence of association with the antibodies. We also predict that if a gene variant is associated with autoantibody positivity, then it becomes a strong candidate as a risk locus for the associated autoimmune disease. In the present report we illustrate that this strategy is successful with the identification of two new candidate genes for Graves' disease susceptibility, *BACH2* and *UBASH3A*.

To investigate the genes involved in autoantibody production, we measured two T1D-associated anti-islet autoantibodies: glutamate decarboxylase (GADA, n = 2,506) and insulinoma-associated antigen 2 (IA-2A, n = 2,498) in plasma samples from T1D cases. In contrast with T1D, Graves' disease is known to be mediated by autoantibodies against the thyroid stimulating hormone receptor (TSHR), which leads to hypothyroidism. However, thyroid peroxidase autoantibodies (TPOA), which are detected in 75% of Graves' disease patients [12], are a sensitive and specific predictor of the disease. TPOA are also correlated with anti-TSHR autoantibodies and Hashimoto's thyroiditis [12]. This direct role of anti-TSHR antibodies in Graves' disease aetiology motivated the measurement, in the same collection of T1D cases, of TPOA (n = 8,300), which was an assay available at a significantly lower cost than the anti-TSHR autoantibody test. To further extend this analysis, we also measured autoantibodies directed against parietal cells (PCA, n = 4,328), a biomarker for autoimmune gastritis and pernicious anaemia. Eighty-six percent of pernicious anaemia patients are estimated to be PCA positive [13].

We combined these four autoantibody measurements (GADA, IA-2A, PCA and TPOA) with available genome-wide genotype data to carry out four distinct genome-wide association (GWA) scans for autoantibody positivity. Outside of the HLA region, we discovered the association of several genes with autoantibody positivity, including evidence for association of the *ABO* blood gene with autoimmunity and also, surprisingly, a strong association of the known autoimmunity gene, *FCRL3*, with IA-2A, but not with T1D. Associations with variants in the HLA region are the subject of a separate paper (Howson *et al.*, *Diabetes*).

## Results

In the 8,506 T1D samples the median age at venepuncture was 13 years, the median age at T1D diagnosis was 8 years and the median time between T1D diagnosis and venepuncture was 5 years. Thirty percent of the blood samples were taken within two years. The distribution of the four antibody measurements is shown in Figure S1. GADA and IA-2A -seropositive cases were at 50% and 59%, respectively (Table 1). Consistent with previous reports [1], time since diagnosis is negatively correlated with GADA and IA-2A positivity (Table 1), perhaps reflecting declining levels of beta-cell antigens and immuno-inflammatory activity following diagnosis [14]. We, therefore, included time since diagnosis (or disease duration) as a covariate in all statistical analysis. After controlling for time since diagnosis, early-onset T1D cases had lower frequencies of GADA and IA-2A (Table 1).

**Table 1:** Sample size and covariates correlated with autoantibody measurements.

Autoantibody	Sample size			OR (*p*-value)						
	n	% pos	GWA	Time since diagnosis†	Age at diagnosis	Women	GADA	IA-2A	PCA	TPOA
GADA	2506	50%	2261	0.66	3.2	1.8	-	NS	NS	2.1
				(6.5e-12)	(5.2e-27)	(1.1e-11)				(1.1e-07)
IA-2A	2498	59%	2254	0.56	2.6	NS	NS	-	NS	NS
				(2.5e-21)	(1.3e-17)					
PCA	4328	10%	2205	1.4	NS	1.7	NS	NS	-	2.9
				(5.9e-07)		(3.5e-07)				(7.8e-17)
TPOA	8300	12%	5781	1.3	NS	2.3	2	NS	2.9	-
				(1.1e-11)		(1.3e-34)	(2.7e-07)		(9.5e-17)	

*p*-values and estimated odds ratios (OR) for the effect of covariates on autoantibody measurements (logistic regression controlling for other significant covariates). The sample size, n, refers to the number of samples with autoantibody data. For time since diagnosis and age at diagnosis, odds ratios are reported for a 10 year difference. GWA indicate the total number of samples genotyped either on the AffymetrixGeneChip Human Mapping 500 K or Illumina 550 K arrays with autoantibody data.

**†:** : Time since diagnosis is highly correlated with patient's age at venepuncture (correlation coefficient *ρ* = 0.85). NS: Non significant (*p*>0.01).

PCA and TPOA frequencies in the T1D samples were 10% and 12%, respectively (Table 1). Age has been shown to have a major effect on TPOA frequency [15]. In our study, time since T1D diagnosis is positively correlated with PCA and TPOA positivity (Table 1), but because this covariate is strongly correlated with age at venepuncture (correlation coefficient 0.85), this observation is most likely a consequence of age. PCA, GADA and TPOA frequencies were higher in women, a result consistent with previous reports of elevated frequency of autoimmune diseases in women [16], [17]. Lastly, and after controlling for other covariates, significant positive correlations were observed between PCA and TPOA, and between TPOA and GADA (Table 1).

### Analysis of the 1q23/FCRL1-FCRL3 region

We found two (non-MHC) associations using a genome-wide significance threshold of, *p* = 5×10^−8^. Firstly, we associated rs4971154 on chromosome 1q23 in exon 5 of the immune-regulatory receptor gene *FCRL1* with IA-2A (*p* = 2.9×10^−11^, estimated odds ratio (OR) = 0.66 for the minor allele C, Table 2). Three other loci in this chromosome region have been associated with autoantibody and/or autoimmune diseases: rs7528684, located in the promoter region of the *FCRL3* gene, was associated with rheumatoid arthritis (RA) and SLE risk, as well as frequency of cyclic citrullinated peptide autoantibodies (CCPA) in Japanese RA patients [18]. rs11264798 located in intron 8 of *FCRL3*, and rs10489678 in *FCRL5*, have been previously associated with Graves' disease [19].

**Table 2:** Autoantibody associations passing a genome-wide association significance threshold in the GWA scan (MHC excluded).

	SNP	Chr	Gene	Alleles	MAF	N	*p*-value	OR [95%CI]
IA-2A	rs4971154	1q23	*FCRL1*	T>C	0.49	970/1385	2.9e-11	0.66 [0.58–0.74]
PCA	rs657152	9q34	*ABO*	G>T	0.35	1768/158	1.15e-13	0.34 [0.25–0.47]

Non-MHC SNPs passing a genome-wide significance threshold *p*<5×10^−8^ when scanning the genome for association with IA-2A, GADA, PCA, and TPOA. *p*-values and odds ratios (OR, estimated for the minor allele and 95% confidence intervals (CI)) were obtained using logistic regression (1 df trend test). We controlled for significant covariates (sex, time since diagnosis and age at bleed). The sample size N indicates the number of autoantibody negative/positive individuals. Antibody status was coded as a binary variable (Methods). Note that we listed the nearest gene, which does not imply that this gene is causal.

To understand the relationship between the TPOA, IA-2A, T1D and Graves' disease associations at the 1q23 locus, we genotyped rs4971154, rs7528684, rs11264798 and rs10489678 in the full T1D case-control collection (up to 10,596 controls and 8,506 T1D cases), as well as in 2,477 Graves' disease patients (the same cohort that was used in [19]). We used stepwise logistic regression to select the most associated SNP for each of the four traits. The alleles, minor allele frequencies and pairwise measures of LD are shown in Table S1. Association results for these SNPs with IA-2A and T1D are shown in Table 3. TPOA and Graves' results are shown in Table 4.

**Table 3:** T1D and IA-2A association results in the 1q23/*FCRL3* gene region.

		IA-2A				T1D			
SNP	Alleles	IA-2A pos	IA-2A neg	OR [95% CI]	*p*	T1D	Controls	OR [95% CI]	*p*
rs4971154	C	0.44	0.54	0.66[0.58–0.74]	2.9e-11	0.48	0.498	0.95[0.91–0.99]	0.013
	TT	429 (0.31)	203 (0.21)	1	-	2231 (0.27)	2508 (0.25)	1	-
	CT	682 (0.49)	489 (0.5)	0.67[0.54–0.82]	6.2e-05	4182 (0.5)	5133 (0.51)	0.92[0.85–0.98]	0.014
	CC	274 (0.2)	278 (0.29)	0.43[0.34–0.55]	2.4e-10	1974 (0.24)	2477 (0.24)	0.9[0.83–0.97]	0.0087
rs7528684	C	0.4	0.5	0.65[0.58–0.74]	8.3e-12	0.44	0.46	0.97[0.93–1.01]	0.09
	TT	514 (0.36)	251 (0.25)	1	-	2669 (0.31)	3151 (0.3)	1	-
	CT	676 (0.47)	519 (0.51)	0.66[0.54–0.82]	2.8e-06	4213 (0.49)	5246 (0.5)	0.96[0.9–1.02]	0.21
	CC	240 (0.17)	246 (0.24)	0.43[0.34–0.55]	4.6e-10	1714 (0.2)	2199 (0.21)	0.93[0.86–1.01]	0.11
rs11264798	C	0.53	0.46	1.42[1.26–1.61]	1.9e-8	0.5	0.49	1.04[1–1.08]	0.06
	GG	307 (0.22)	287 (0.29)	1	-	2148 (0.25)	2670 (0.26)	1	-
	CG	708 (0.5)	501 (0.51)	1.41[1.14–1.73]	0.0057	4215 (0.5)	5066 (0.5)	1[0.96–1.1]	0.35
	CC	397 (0.28)	199 (0.2)	2.02[1.6–2.6]	1.8e-07	2121 (0.25)	2381 (0.24)	1.1[1–1.2]	0.014
rs10489678	A	0.17	0.21	0.77[0.66–0.89]	6.6e-4	0.18	0.19	0.95[0.91–1.01]	0.078
	GG	967 (0.69)	625 (0.63)	1	-	5656 (0.67)	6623 [0.65)	1	-
	AG	381 (0.27)	313 (0.32)	0.75[0.62–0.91]	0.0091	2485 (0.29)	3124 (0.31)	0.93[0.87–0.99]	0.028
	AA	49 (0.035)	49 (0.05)	0.63[0.41–0.96]	0.036	308 (0.036)	368 (0.036)	0.98[0.84–1.15]	0.8

Genotypic and allelic IA-2A/T1D association results for the four SNPs in the 1q23/*FCRL3* region genotyped in the full JDRF/WT T1D case control collection. For each SNP the first row indicates the minor allele for which the odds ratio (OR) is estimated (95% confidence intervals (CI) is shown between brackets). IA-2A association tests include age at venepuncture, age at T1D onset and region of origin as covariates. T1D tests only include region of origin. For the genotypic association the most common homozygous group was taken as the reference. Numbers in parenthesis indicate the frequency of this genotype or allele.

**Table 4:** T1D, TPOA, and Graves' disease associations for SNPs genotyped in the Graves's disease cohort.

SNP	Chr.	Gene	Alleles	*p* T1D	OR T1D	*p* Graves	OR Graves	*p* TPOA	OR TPOA
rs2476601	1p13.2	*PTPN22*	C>T	2.1e-111	1.9–2.1	2.2e-14	1.4–1.7	2.1e-05	1.2–1.4
rs11264798	1q23.1	*FCRL3*	G>C	-	-	0.0017	0.83–0.96	0.00035	0.74–0.94
rs4971154	1q23.1	*FCRL1*	T>C	0.013	0.91–0.99	0.0011	1–1.2	0.0033	1.1–1.3
rs7528684	1q23.1	*FCRL3*	T>C	-	-	0.0039	1–1.2	0.02	1–1.2
rs10489678	1q23.1	*FCRL5*	G>A	-	-	-	-	-	-
rs1990760	2q24.2	*IFIH1*	A>G	2.2e-14	0.81–0.89	-	-	-	-
rs3087243	2q33.2	*CTLA4*	C>T	2.3e-17	0.79–0.87	1e-21	0.66–0.76	0.0011	0.73–0.95
rs2069762	4q27	*IL2-IL21*	T>G	5.4e-07	0.84–0.93	-	-	0.0045	1.1–1.3
rs2069763	4q27	*IL2-IL21*	G>T	7.4e-08	1.1–1.2	0.026	0.85–0.99	-	-
rs6822844	4q27	*IL2-IL21*	G>T	0.034	0.89–1	-	-	-	-
rs6897932	5p13.2	*IL7R*	C>T	0.0026	0.89–0.98	-	-	-	-
rs6887695	5q33	*IL12B*	G>C	-	-	-	-	-	-
rs11755527	6q15	*BACH2*	C>G	3.1e-08	1.1–1.2	0.0062	1–1.2	9.7e-07	1.2–1.4
rs1738074	6q25.3	*TAGAP*	G>A	0.00051	0.89–0.97	0.049	0.86–1	-	-
rs11594656	10p15.1	*IL2RA*	T>A	2e-06	0.84–0.94	0.00028	0.78–0.93	-	-
rs689	11p15.5	*INS*	A>T	5.2e-196	0.38–0.49	-	-	-	-
rs11175593	12q12	*LRRK2*	C>T	0.028	1–1.4	-	-	-	-
rs2292239	12q13.2	*ERBB3*	C>A	2.9e-27	1.2–1.3	-	-	-	-
rs662739	12q24.31	*SPPL3*	G>A	-	-	0.0086	0.84–0.97	0.011	1–1.2
rs3184504	12q24.12	*SH2B3*	C>T	2e-38	1.3–1.4	-	-	0.003	1.1–1.3
rs7171171	15q14	*RASGRP1*	A>G	2.3e-7	1.09–1.2	-	-	0.0004	0.71–0.91
rs12708716	16p13.13	*CLEC16A*	A>G	5e-14	0.8–0.89	-	-	-	-
rs478582	18p11.21	*PTPN2*	T>C	2.8e-12	0.82–0.9	0.011	0.84–0.98	-	-
rs763361	18q22.2	*CD226*	C>T	1.3e-09	1.1–1.2	0.045	1–1.2	-	-
rs3788013	21q22.3	*UBASH3A*	C>A	1e-07	1.1–1.2	0.00024	1.1–1.2	0.00099	1.1–1.3

T1D, Graves' disease, and TPOA association results for the set of SNPs that were typed in the Graves' disease cohort (2,477 cases). 95% confidence intervals are shown for the minor allele (1 df linear trend tests stratified by region of origin in the UK). When *p*>0.05, 95% confidence intervals for the odds ratio (OR) and *p*-values are not shown. We list the closest gene that may not be the causal one. In particular recent data (Cooper *et al.*, submitted) show that the rs689732/*IL7R* is secondary to a SNP located in the *CAPSL* gene.

We found that the IA-2A association in T1D cases was fully accounted for by the *FCRL3* SNP rs7528684 (*p* = 8.3×10^−12^, OR = 0.65 for the minor allele C, Table 3), which added to other single SNP models in the stepwise regression (*p*<0.01). rs7528684 was denoted as 169C→T in [18], where Kochi *et al.* reported that the RA and SLE risk allele rs7528684-C was also associated with increased *FCRL3* expression and higher CCPA frequency in RA patients. In our European sample sets, the minor allele of rs7528684 is C and was strongly *negatively* associated with IA-2A positivity, a direction of effect opposed to the previously reported CCPA result. We checked this result thoroughly by resequencing both alleles in four selected samples and verified the initial data (Y. Kochi, personal communication), but found no errors. We also replicated the IA-2A/rs7528684 in 3,897 affected siblings from multiplex families (Methods) and found consistent evidence of IA-2A negative association (*p* = 7.6×10^−9^, OR = 0.69).

The TPOA and Graves' disease associations showed the same pattern of association, whereby a single (although different) SNP, rs11264798, alone explained both associations (*p* = 3.5×10^−4^ for TPOA and *p* = 1.7×10^−3^ for Graves' disease, Table 4). The SNP rs11264798 added to other single SNP models in a stepwise regression (*p*<0.01). The discrepancy between the TPOA and IA-2A results suggests that the causal variants underlying these two associations are distinct. We confirmed this hypothesis using a formal statistical test [20] (*p* = 0.004 against the null hypothesis of a single causal variant).

In contrast with the IA-2A and TPOA/Graves' disease result, there was no evidence for T1D case-control association in this region: *p* = 0.09 for rs7528684 and *p* = 0.06 for rs11264798 in 10,010 controls and 8,327 T1D cases, estimated OR = 0.97 and 1.04, respectively, for the minor alleles (Table 3). To evaluate this result further we genotyped both SNPs in a set of 8,038 T1D trios (3,598 multiplex families, see Methods), obtaining weak evidence of T1D association (*p* = 0.026 for rs7528684 and *p* = 0.04 for rs11264798, with relative risks 0.95 and 1.04, respectively). Taken together, these data suggest that the minor allele C of rs7528684 is very weakly protective for T1D (combined *p* = 0.0013, OR = 0.95) and the minor allele C of rs11264798 increases T1D risk very slightly (combined *p* = 0.0014, OR 1.04). The rs11264798-C minor allele is protective for TPOA, but is also in LD with the rs7528684-T major allele associated with IA-2A positivity.

### PCA association at the 9q34/ABO locus

PCA positivity was associated with rs657152 (G>T) on chromosome 9q34, in intron 1 of the blood group gene *ABO* (*p* = 1.15×10^−13^, OR = 0.35 for the minor allele T, Table 2). rs657152-G is a marker for the ABO blood group O in Caucasian individuals (using blood group frequency estimates based on rs6872889 in [21], which is a proxy for rs657152, HapMap r^2^ = 0.93). We found a departure from the linear trend test assumption for this SNP (*p* = 0.02 when we compared a 2 degree-of-freedom genotype effect model to the standard 1 degree-of-freedom linear trend test). Taking the most common genotype GG as reference the estimated odds ratios were 0.26 for GT (95% CI: 0.17–0.39) and 0.23 for TT (95% CI: 0.11–0.49). Therefore in this dataset the observed PCA association is essentially a consequence of the elevated PCA frequency in the GG genotype group, which is closely correlated to the ABO blood group O.

The major allele G at this SNP, associated with higher PCA frequency in T1D cases, has also been associated with multiple traits including higher circulating levels of soluble intercellular adhesion molecule 1 (sICAM-1, [21]) and E-selectin [22], higher gastric ulcer risk [23] and lower pancreatic cancer risk [24], indicating that this blood group determinant enzyme has pleiotropic effects. We found no association of this SNP with T1D (*p* = 0.268 in 7,240 controls and 5,817 T1D cases). We investigated whether other sICAM associated SNPs in a different chromosome region (19p13, [21]) showed association with PCA (Table S2), but found no evidence supporting this.

At the *FUT2* gene locus, the A allele of rs601338A>G (X143W/se428) prevents the secretion of ABO antigens in the gut and in saliva. The homozygous genotype AA has recently been associated with susceptibility for Crohn's disease [25], [26] and to T1D (DJS, JMMH, JAT, unpublished, www.t1dbase.org). We therefore tested a potential PCA association with this SNP. We found unconvincing evidence of PCA association: *p* = 0.077 linear trend test in 437 PCA positive and 3,697 PCA negative samples, estimated OR = 0.88 (95% CI: 0.77–1.01) for the G allele. Taking the AA non-secretor group as reference, a genotype association analysis suggests that the estimated ORs are not significantly different for the AG (OR = 0.81, 95% CI: 0.64–1.01) and GG (OR = 0.79, 95% CI: 0.796–1.05) genotype groups. Therefore, while the evidence is unconvincing for PCA, this recessive model of increased risk for non-secretor AA genotype group is consistent with the T1D and Crohn disease associations.

### Autoantibody association at T1D loci

We then used additional genotyping data in the full T1D case collection to investigate autoantibody associations at 64 known T1D-associated SNPs in 52 distinct chromosome regions ([27], www.t1dbase.org). Owing to higher prior belief that these SNPs are autoantibody associated we used a less stringent threshold (*p*≤0.01) corresponding here to a false discovery rate of 16% (Benjamini-Hochberg estimation procedure [28]). We identified 13 independent associations, which included two SNPs associated with two distinct autoantibodies each (Table 5). The most significant finding was the TPOA association with the *BACH2* T1D-associated SNP rs11755527 (C>G, *p* = 9.7×10^−7^, OR = 1.27). Eight out of these 13 associations involved TPOA. The association of TPOA with *CTLA4* in T1D cases has been reported previously [16], and here, we extend support for this finding. We did not replicate a previously published GADA/*PTPN22* interaction [29]. We also did not obtain evidence of association with any of the autoantibodies (*p*>0.05), including IA-2A and GADA, with *INS*, which shows the strongest association with T1D outside of the MHC region.

**Table 5:** Autoantibody associations at published T1D associated loci.

				T1D	GADA		IA-2A		PCA		TPOA	
SNP	Chr	Gene	All	OR[95% CI]	*p*	OR[95% CI]	*p*	OR[95% CI]	*p*	OR[95% CI]	*p*	OR[95% CI]
rs2476601	1p13.2	*PTPN22*	C>T	2[1.88–2.13]	-	-	-	-	-	-	2.07e-05	1.3[1.15–1.46]
rs1990760	2q24.2	*IFIH1*	A>G	0.848[0.81–0.89]	-	-	0.0036	1.2[1.06–1.36]	0.000699	0.772[0.66–0.9]	-	-
rs7574865	2q32.3	*STAT4*	G>T	1.1[1.04–1.15]	-	-	-	-	-	-	0.01	1.16[1.04–1.3]
rs3087243	2q33.2	*CTLA4*	C>T	0.829[0.79–0.87]	-	-	-	-	-	-	0.00113	0.839[0.75–0.93]
rs2069762	4q27	*IL2*	T>G	0.889[0.85–0.93]	-	-	-	-	-	-	0.00452	1.17[1.05–1.3]
rs11755527	6q15	*BACH2*	C>G	1.13[1.08–1.17]	-	-	-	-	-	-	9.67e-07	1.27[1.15–1.4]
rs12722495	10p15.1	*IL2RA*	A>G	0.618[0.57–0.67]	0.00658	0.734[0.59–0.92]	-	-	-	-	-	-
rs3184504	12q24.12	*SH2B3*	C>T	1.32[1.27–1.38]	0.00175	1.21[1.07–1.36]	-	-	-	-	0.003	1.16[1.05–1.28]
rs7171171	15q14	*RASGRP1*	A>G	1.14[1.09–1.2]	-	-	-	-	-	-	0.0004	0.8[0.71–0.91]
rs4788084	16p11.2	*IL27*	G>A	0.879[0.84–0.92]	-	-	0.00305	1.21[1.07–1.37]	-	-	-	-
rs3788013	21q22.3	*UBASH3A*	C>A	1.12[1.07–1.17]	-	-	-	-	-	-	0.000991	1.18[1.07–1.29]

Tests of 64 confirmed T1D susceptibility SNPs (located in 52 distinct chromosome regions, see www.t1dbase.org) for autoantibody association (*p*≤0.01, false discovery rate 16%). SNPs without any positive autoantibody association are not shown. The full JDRF/WT T1D case-control collection was used (up to 10,596 controls and 8,506 T1D cases, n = 2,506 for GADA, n = 2,498 for IA-2A, n = 4,328 for PCA and n = 8,300 for TPOA). *p*-values and odds ratio (OR) and 95% confidence interval (CI) for the minor allele are computed using logistic regressions independently for each SNP (1 df trend test) controlling for significant covariates (sex, time since diagnosis and age at diagnosis). Autoantibody status was coded as a binary variable (Methods).

To replicate both IA-2A associations in an independent collection, we genotyped the *IL27* SNP rs4788084 and *IFIH1* SNP rs1990760 in the 3,897 T1DGC affected sibling samples with IA-2A data (Methods). Both SNPs convincingly replicated the initial IA-2A results: two-tailed *p* = 0.0074, 8.4×10^−4^ for *IFIH1/*rs1990760, *IL27/*rs4788084, respectively, with the direction of effect consistent in both cases with the initial finding (estimated OR = 1.13 for *IFIH1* and 1.17 for *IL27*).

### Graves' disease association for newly identified TPOA loci

TPOA are commonly detected in Graves' disease patients and, therefore, the TPOA- associated SNPs located in the genes *RASGRP1*, *UBASH3A* and *BACH2* that have not been previously tested for Graves' disease association are strong Graves' disease candidates. To investigate this hypothesis we genotyped these three SNPs in 2,477 Graves' disease cases (Table 4). We obtained *p* = 2.4×10^−4^, OR = 1.14 for the *UBASH3A* C>A SNP rs3788013 and *p* = 6.2×10^−3^, OR = 1.11 for the *BACH2* C>G SNP rs11755527. For both SNPs the minor allele is the risk allele for Graves' disease and T1D and was associated with higher positivity for TPOA. No evidence of Graves' disease association was found for rs7171171 in *RASGRP1* (*p*>0.05).

### Analysis of additional autoimmune associated findings

Given the positive findings in T1D associated loci we extended our analysis to 135 SNPs in 100 autoimmune-associated loci [30], [31], [32]. Each SNP was tested for association with TPOA, GADA, PCA and IA-2A using *p*≤0.01 as a threshold (false discovery rate of 27%, Table S3). We found five additional associations: 2q37/*PDCD1*-IA-2A, 3p14.3/*PXK*-GADA, 5q33.3/*IL12B*-TPOA, 12q12/*LRRK2*-TPOA, 12q24.31/*SPPL3*-TPOA. We genotyped the full T1D case-control collection as well as the set of Graves' disease cases to validate the three TPOA findings, but none of them replicated for TPOA, or provided convincing evidence of Graves' disease or T1D association (*p*>0.05).

## Discussion

The combination of genome-wide genotyping data with autoantibody measurements and case-control data for T1D and Graves' disease has enabled the discovery of several new genetic associations with autoantibody positivity and disease traits. We conclude that the nine loci (*FCRL3*, *RASGRP1*, *SH2B3*, *STAT4*, *BACH2*, *UBASH3A*, *IL2*, *PTPN22*, and *CTLA4*) associated with TPOA, which is not a T1D anti-islet autoantibody, may have general effects in adaptive immunity, in the complex interactions between antigen presenting cells and T cells leading to antibody-producing plasma B cells. Consistent with this general role in autoimmunity, six out of nine TPOA loci were associated with Graves' disease (except rs2069762 in *IL2*, rs3184504 in *SH2B3* and rs7171171 in *RASGRP*, Table 5), including both newly identified Graves' disease loci *BACH2* and *UBASH3A*. For these six TPOA/Graves' disease-associated SNPs, the Graves' disease risk allele is also the one associated with elevated frequency of TPOA. Lastly, four of the nine TPOA associated SNPs (in *SH2B3*, *CTLA4*, *BACH2* and *UBASH3A*) have also been associated with celiac disease [33] (but not the SNPs in *RASGRP1*, *STAT4*, *PTPN22*, *IL2* [20] and *FCRL3* [33]). We note that the Graves' disease associated thyroglobulin (TG) gene region, was not associated with TPOA (*p*>0.01 at all SNPs within 300 kb of the TG gene). To further understand the association of genes with autoantibodies in T1D and in Graves' disease the measurement of TSHR autoantibodies will be informative.

After controlling for time since diagnosis we found that the patients' age at T1D diagnosis was positively correlated with the presence of GADA and IA-2A, such that patients diagnosed at a younger age are less likely to be IA-2A or GADA positive. This result is consistent with other studies that found that GADA positivity was associated with older age at diagnosis [34], [35]. Our finding suggests that earlier onset T1D involves pathogenesis directed towards autoantigens other than GADA/IA-2A, and/or beta-cell destruction is so profound in these children that they lose their autoantibodies very rapidly owing to extensive removal of islet antigens following T1D onset.

Owing to the fact that the plasma samples in this study were collected a median time of five years following diagnosis, with 30% within 2 years of diagnosis, we cannot exclude the possibility that the same GWA conducted using autoantibody data closer or prior to T1D onset might yield different results. Nevertheless, the prevalences of GADA and IA-2A in our study (Table 1) are consistent with previously reported measurements in paediatric patients at T1D diagnosis [35], [36], [37], [38]. The misclassification of autoantibody status in T1D cases may lower the statistical power but it is highly unlikely to generate false positive genetic associations. Hence, the convincing association results identified in this study (Table 2, Table 3, Table 4, Table 5) indicate that these measurements are valid.

Our estimated TPOA frequencies are consistent with a recent large scale study [17] which found 8.8% of T1D children aged less than 12 years to be TPOA positive. However, the strong effect of age, and the use in other studies of different assays with variable sensitivity, complicates a comparison of autoantibody frequency with healthy control groups. A previous study [39] found 2.6% of Finnish school children and 0.4% of Russian school children to be TPOA positive but another Swedish study [40] found 11.3% of 12 year old children to be TPOA positive. Our frequency of PCA positivity in plasma samples from T1D cases of 10% is comparable to previous reports of PCA frequencies in T1D diagnosed under age 30 years (9% in [41]), but higher in that the 2.2% in population controls aged 21–30 years [41].

Among the four autoantibodies we considered, the absence of correlations between IA-2A and GADA/PCA/TPOA (Table 1) suggests the involvement of distinct genes and pathways for IA-2A. Moreover, for the T1D-associated SNPs located in *IFIH1* and *IL27*, the T1D risk allele is associated with reduced IA-2A positivity (Table 5). This result is different from the TPOA/T1D associations for which, in six out of eight cases, the T1D risk allele is also the allele associated with increased TPOA positivity (rs7171171 in *RASGRP1* and rs2069762 in *IL2* being the exceptions, see Table 5). This pattern has been reported previously between IA-2A and the T1D associated HLA-A*24 allele [36] and confirmed by our recent analyses (JMMH, JAT, *Diabetes*).

The 1q23*/FCRL3* association data highlight the complexity of this autoimmune locus, which has previously been associated with SLE, RA and Graves' disease. Our results show that two distinct associations co-localize in this chromosome region. Firstly, the SNP rs7528684-C is associated with SLE and RA risk, CCPA positivity in RA patients, but is negatively associated with IA-2A positivity in T1D. These associations, which are in opposite directions for IA-2A compared to the other autoimmune traits, contrast with the consistency observed for the *PTPN22* and *CTLA4* variants, for which the risk allele is consistently the same across multiple autoimmune diseases (in particular Graves' disease, T1D and TPOA in T1D patients, see Table 4). Secondly, the SNP rs11264798-G is independently associated with Graves' disease, as well as with TPOA positivity in T1D patients. The three autoantibody associations in this chromosome region (with CCPA, IA-2A and TPOA) indicate that this locus is involved in the breakdown of self-tolerance and autoantibody production. On the other hand, the effect on T1D risk is not strong (combined case-control and family *p* = 0.001). Owing to the involvement of this region in multiple autoimmune disorders the prior belief that this locus is T1D associated is high. Therefore, the T1D association result could be real, but the effect size very small (estimated odds ratio 1.05). One explanation for these highly significant results in terms of IA-2A association is that the autoimmune disease-associated allele, C (of SNP rs7528684) is affecting anti-IA-2A T cells responses in a different way to autoantibody responses to this antigen [42]. The FCRL3 molecule could be affecting T regulatory cell development or function [43].

The *ABO* gene encodes a glycosyltransferase which is expressed in multiple human tissues. It could affect glycosylation, and therefore function or antigenicity of a wide range of molecules, in particular parietal cells antigens in the gastro-intestinal mucosal lining [44]. This blood group O, associated with increased PCA frequency, is also associated with increased frequency of gastric ulcers [23], a condition frequently caused by long-standing *Helicobacter pylori* infection. ABO blood groups are not associated with the presence of *H. pylori* [45] but the blood group O has been associated with increased inflammatory response to this bacterium [46]. A plausible hypothesis for the ABO-PCA association is that the inflammation caused by *H. pylori* can not only result in gastric ulcers, but can also initiate an autoimmune reaction directed against parietal cells. However, previous reports do not support an increased pernicious anaemia risk for individuals with the ABO blood group O [47], [48], [49], which indicates that the role of *ABO* in progression from PCA to pernicious anaemia is not straightforward.

Finally, we note that the majority of T1D regions did not associate with autoantibody positivity. Many of these chromosome regions contain genes of unknown function with no obvious candidate genes. It will be informative to continue to compare genetic associations from other diseases and traits (such as autoantibodies analysed here and other serum analytes, such as soluble CD25 [50]), to identify which of these newly-mapped, unexplored T1D loci are involved in certain pathways. Our current results place the candidate genes *SH2B3*, *CTLA4*, *BACH2* and *UBASH3A* at the very heart of the immune response in the pathogenesis of both T1D and celiac disease.

## Material and Methods

### T1D cases and autoantibody measurements

9,381 T1D case samples (DNA and plasma) were available as part of the Juvenile Diabetes Research Foundation/Wellcome Trust Diabetes and Inflammation Laboratory type 1 diabetes case GRID (Genetic Resource Investigating Diabetes) collection (white British individuals diagnosed before age 17). Autoantibodies were measured in plasma for a subset of them (GADA, n = 2,506, IA-2A, n = 2,498, PCA, n = 4,328, TPOA, n = 8,300).

Presence of each of the autoantibodies (IA-2A, GADA) in the type 1 diabetes cases was tested using plasma stored at −80°C in aliquots. Autoantibodies to GAD and IA-2A were measured in the Department of Clinical Science at North Bristol, University of Bristol, using a radioimmunoassay [51]. GADA sensitivity was 86% and specificity 99%, while IA-2A sensitivity was 72% and specificity 93% in the Diabetes Antibody Standardization Program 2005 (23). Presence of GADA and IA-2A was taken as above 14 and 6 WHO Units/ml, respectively, which corresponds to the 97.5^th^ percentile of the distribution of these autoantibodies in 2,860 school children from Oxford, UK [51].

TPOA and PCA were measured by the Department of Clinical Biochemistry, University of Cambridge with, respectively, a PLATO processor ELISA immunoassay (Phadia, Milton Keynes, UK) using recombinant TPO antigen standardised against the National Institute of Biological Standards and Controls standard serum 66/387 in which the positivity threshold for TPOA was 85 IU/ml, and ELISA manufactured by Phadia and analysed on a QIAGEN Plato III platform in which the threshold for presence of PCA was 10 U/ml.

### Type 1 diabetes affected sib-pair families

To follow-up on a T1D association observed in the case-control data we genotyped 3,598 affected sib-pair families. The majority was available through the Type 1 Diabetes Genetics Consortium (T1DGC; http://www.t1dgc.org; http://www-gene.cimr.cam.ac.uk/todd/dna-refs.shtml). Of these T1DGC families: 237 were from the T1DGC Asia–Pacific region, 580 from T1DGC North-America and 1,103 from T1DGC-Europe. In addition, 354 families originated from the UK-Warren collection, 298 from HBDI (http://www.ndriresource.org/NDRI_Initiatives/HBDI/36/) and 1,026 from Finland. These samples were used for T1D association testing and IA-2A was the only autoantibody data available. As for the T1D case control samples, autoantibodies were measured on average several years after T1D diagnosis.

### Graves' disease cases

To test for association with Graves' disease, a total of 2,477 unrelated white ethnic group, British Graves' disease patients were recruited as part of the autoimmune thyroid disease UK National Collection. Patients were recruited from centres across England and Wales including Birmingham, Bournemouth, Cambridge, Cardiff, Exeter, Leeds, Newcastle and Sheffield. All recruiting centres used standard clinical criteria to diagnose Graves' disease to avoid any clinical heterogeneity. These samples were solely used to test for Graves' disease association and no autoantibody data was available.

### Control samples

To test for disease association (T1D and Graves' disease) control samples consisted of individuals from the British 1958 Birth Cohort and UK blood donors National Health Service Blood and Transplant [19]. Controls were matched to cases using place of recruitment for each of 12 geographical regions of Great Britain (Southern England, South-Western England, South-Eastern England, Eastern England, London, Midlands, Wales, North-Eastern England, North Midlands, East and West Ridings, Northern England, Scotland). All cases and controls were of self-reported white ethnicity. All DNA samples (T1D cases, Graves' cases and controls) were collected with approval from the relevant research ethics committee and written informed consent was obtained from the participants or their guardians. No autoantibody data were available for control samples.

### Genotyping

Most of T1D cases with autoantibody data were genotyped previously using the Affymetrix 500K mapping array [19] or the Illumina 550K array [27]. We combined data from both arrays using an imputation procedure [27] to carry out a genome-wide scan for autoantibody association (n = 2,261 for GADA, n = 2,254 for IA-2A, n = 2,205 for PCA, n = 5,781 for TPOA).

### Statistical analysis

Association between variants and autoantibodies were tested using regression models, treating positive autoantibody status as a binary outcome and using a one-degree-of-freedom trend test (log-scale additive disease model). Significant covariates were included (sex, time since diagnosis and age at bleed, age at diagnosis of T1D). Similar analysis was performed to test for T1D and Graves' disease association. Geographical region was included as a confounder in all logistic regression models. Statistical analyses were performed using the R statistical software.

## Supporting Information

Figure S1 Histograms of the four log-transformed autoantibody measurements : IA-2A, GADA, PCA, TPOA. The vertical red dashed lines indicate the positivity cut-offs. Details on experimental design are provided in Material and Methods.
(PDF)

Table S1 Alleles (X>Y, where Y is the minor allele), minor allele frequency (MAF) and pairwise pattern of linkage disequilibrium in UK controls for the four SNPs with published autoantibody and/or disease association in the *FCRL3* chromosome region. The notation x/y refers to the standard r^2^/D′ values for pairwise measures of linkage disequilibrium.
(PDF)

Table S2 PCA and sICAM association *p* -values [21] for SNPs associated with sICAM at a genome-wide significance levels and not located in or near the ABO gene. SNPs are taken from Table 1 in [21]. All SNPs are located in the 19p13.2 chromosome region.
(PDF)

Table S3 Genome-wide scan *p*-values and estimated minor allele odds ratios (OR) for GADA, IA-2A, PCA, TPOA and T1D associations for 135 SNPs associated with other autoimmune disorders [30], [31] (excluding SNPs in the HLA locus or that are only T1D associated). When available, we used follow-up genotyping data in the maximum available sample size in the JDRF/WT T1D case control collection. The symbol * indicates that the SNP has been associated with systemic lupus erythematosus (see [31], [32]). Only *p*-values and odds ratios more significant than 0.01 are shown (false discovery rate of 27%).The column Gene only refers to the nearest, or most likely candidate gene, and in most cases the causal gene may actually differ.
(PDF)
