# Supplementary material for: Genome-Wide Association Analysis of Autoantibody Positivity in Type 1 Diabetes Cases
Source: PLoS Genet. 2011 Aug 4;7(8):e1002216. doi: 10.1371/journal.pgen.1002216 (PMC3150451; doi:10.1371/journal.pgen.1002216)
Supplement: Figure S1 — Histograms of the four log-transformed autoantibody measurements : IA-2A, GADA, PCA, TPOA. The vertical red dashed lines indicate the positivity cut-offs. Details on experimental design are provided in Material and Methods. (PDF) [file pgen.1002216.s001.pdf]

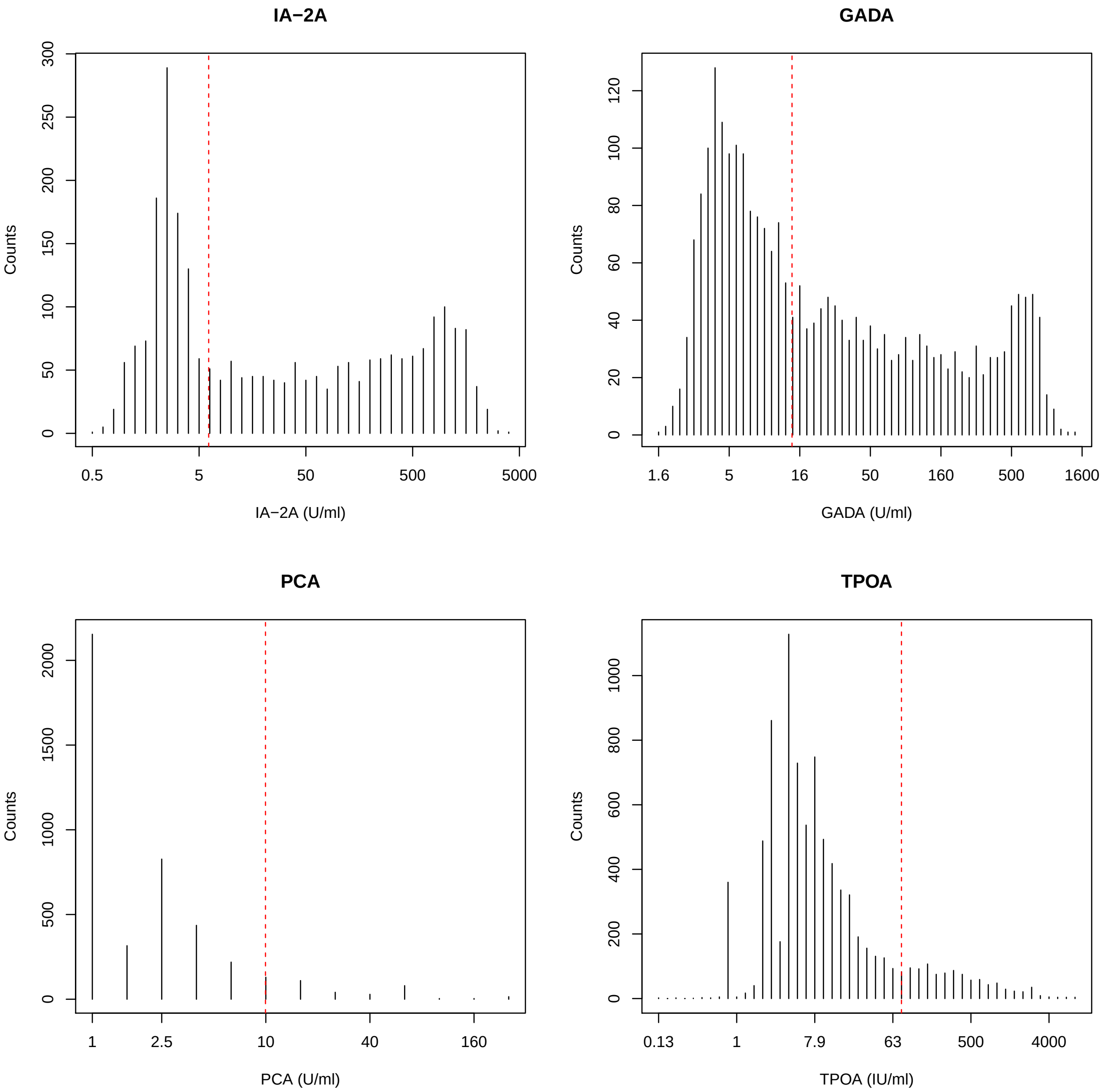

Figure S1: Histograms of the four log-transformed autoantibody measurements : IA-2A, GADA, PCA, TPOA. The vertical red dashed line indicates the positivity cutoff. Details on experimental design are provided in Material and Methods.
