# Supplementary material for: Genome-Wide Association Analysis of Autoantibody Positivity in Type 1 Diabetes Cases
Source: PLoS Genet. 2011 Aug 4;7(8):e1002216. doi: 10.1371/journal.pgen.1002216 (PMC3150451; doi:10.1371/journal.pgen.1002216)
Supplement: Table S1 — Alleles (X>Y, where Y is the minor allele), minor allele frequency (MAF) and pairwise pattern of linkage disequilibrium in UK controls for the four SNPs with published autoantibody and/or disease association in the FCRL3 chromosome region. The notation x/y refers to the standard r2/D′ values for pairwise measures of linkage disequilibrium. (PDF) [file pgen.1002216.s002.pdf]

	rs7528684	rs11264798	rs10489678	rs4971154
Alleles	T>C	G>C	G>A	T>C
MAF	0.45	0.49	0.19	0.49
rs7528684	-	0.78/0.99	0.28/0.99	0.53/0.79
rs11264798	-	-	0.22/1	0.51/0.74
rs10489678	-	-	-	0.21/0.93
rs4971154	-	-	-	-

Table S1: Alleles (X>Y, where Y is the minor allele), minor allele frequency (MAF) and pairwise pattern of linkage disequilibrium in UK controls for the four SNPs with published autoantibody and/or disease association in the *FCRL3* chromosome region. The notation x/y refers to the standard r^2/D' values for pairwise measures of linkage disequilibrium.
