# Supplementary material for: Genome-Wide Association Analysis of Autoantibody Positivity in Type 1 Diabetes Cases
Source: PLoS Genet. 2011 Aug 4;7(8):e1002216. doi: 10.1371/journal.pgen.1002216 (PMC3150451; doi:10.1371/journal.pgen.1002216)
Supplement: Table S2 — PCA and sICAM association p -values [21] for SNPs associated with sICAM at a genome-wide significance levels and not located in or near the ABO gene. SNPs are taken from Table 1 in [21]. All SNPs are located in the 19p13.2 chromosome region. (PDF) [file pgen.1002216.s003.pdf]

SNP	p-value PCA	p-value sICAM (from [21])
rs10409243	0.173	5.4e-15
rs1799969	0.974	3.6e-47
rs2116941	0.169	3.1e-11
rs2278442	0.657	3.1e-11
rs2304237	0.766	3.9e-13
rs281440	0.31	1.5e-16
rs5498	0.55	4.8e-25
rs7256672	0.62	6.3e-10
rs7258015	0.886	2.1e-12
rs8111930	0.387	1.3e-18

Table S2: PCA and sICAM association P-values [21] for SNPs associated with sICAM at a genome-wide significance levels and not located in or near the *ABO* gene. SNPs are taken from Table 1 in [21]. All SNPs are located in the 19p13.2 chromosome region.
