# Supplementary material for: Genome-Wide Association Analysis of Autoantibody Positivity in Type 1 Diabetes Cases
Source: PLoS Genet. 2011 Aug 4;7(8):e1002216. doi: 10.1371/journal.pgen.1002216 (PMC3150451; doi:10.1371/journal.pgen.1002216)
Supplement: Table S3 — Genome-wide scan p-values and estimated minor allele odds ratios (OR) for GADA, IA-2A, PCA, TPOA and T1D associations for 135 SNPs associated with other autoimmune disorders [30], [31] (excluding SNPs in the HLA locus or that are only T1D associated). When available, we used follow-up genotyping data in the maximum available sample size in the JDRF/WT T1D case control collection. The symbol * indicates that the SNP has been associated with systemic lupus erythematosus (see [31], [32]). Only p-values and odds ratios more significant than 0.01 are shown (false discovery rate of 27%).The column Gene only refers to the nearest, or most likely candidate gene, and in most cases the causal gene may actually differ. (PDF) [file pgen.1002216.s004.pdf]

				GADA		I-A2A		PCA		TPOA		T1D	
SNP	Chr	Closest Gene	Alleles	P	OR	P	OR	P	OR	P	OR	P	OR
rs3890745	1p36.32	*MMEL1*	T>C	-	-	-	-	-	-	-	-	-	-
rs186037*	1p36.12	*C1QA*	A>G	-	-	-	-	-	-	-	-	-	-
rs11805303	1p31.3	*IL23R*	C>T	-	-	-	-	-	-	-	-	-	-
rs11209032	1p31.3	*IL23R*	G>A	-	-	-	-	-	-	-	-	-	-
rs3790565*	1p31.3	*IL12RB2*	T>C	-	-	-	-	-	-	-	-	-	-
rs672797	1p31.1	*AL158218.11*	A>C	-	-	-	-	-	-	-	-	-	-
rs2476601*	1p13.2	*PTPN22*	G>A	-	-	-	-	-	-	2.1e-05	1.2	2.1e-111	2
rs11264798	1q23.1	*FCRL3*	G>C	-	-	2.7e-08	1.4	-	-	0.00035	0.84	-	-
rs7528684*	1q23.1	*FCRL3*	T>C	-	-	1.1e-11	0.66	-	-	-	-	-	-
rs2794520*	1q23.2	*CRP*	C>T	-	-	-	-	-	-	-	-	-	-
rs2274910	1q23.3	*ITLN1*	C>T	-	-	-	-	-	-	-	-	-	-
rs1079109*	1q23.3	*HSPA6*	C>T	-	-	-	-	-	-	-	-	-	-
rs12118043*	1q23.3	*FCGR2B*	C>A	-	-	-	-	-	-	-	-	-	-
rs10489185*	1q24.2	*SELP*	C>A	-	-	-	-	-	-	-	-	-	-
rs9286879	1q24.3	*FASLG*	A>G	-	-	-	-	-	-	-	-	-	-
rs10127728*	1q25.1	*TNFSF4*	G>T	-	-	-	-	-	-	-	-	-	-
rs10798269*	1q25.1	*RPL26P11*	G>A	-	-	-	-	-	-	-	-	-	-
rs2811558*	1q25.3	*NMNAT2*	G>T	-	-	-	-	-	-	-	-	7.9e-05	0.91
rs2702180	1q25.3	*NCF2*	T>C	-	-	-	-	-	-	-	-	0.0027	0.93
rs10801047	1q31.2	*RP11-309H21.1*	T>A	-	-	-	-	-	-	-	-	-	-
rs2816316	1q31.2	*RGS1*	A>C	-	-	-	-	-	-	-	-	0.00011	0.9
rs11584383	1q32.1	*KIF21B*	T>C	-	-	-	-	-	-	-	-	-	-
rs3024505*	1q32.1	*IL10*	G>A	-	-	-	-	-	-	-	-	4.7e-10	0.83
rs12139795*	1q32.2	*CR2*	A>G	-	-	-	-	-	-	-	-	-	-
rs9782955*	1q42.3	*LYST*	C>T	-	-	-	-	-	-	-	-	0.003	0.92
rs780094	2p23.3	*GCKR*	C>T	-	-	-	-	-	-	-	-	-	-
rs13385731*	2p22.3	*RASGRP3*	T>C	-	-	-	-	-	-	-	-	-	-
rs13015714	2q12.1	*IL18R1*	T>G	-	-	-	-	-	-	-	-	-	-
rs917997	2q12.1	*IL18RAP*	C>T	-	-	-	-	-	-	-	-	-	-
rs1990760*	2q24.2	*IFIH1*	T>C	-	-	0.0036	0.85	0.0007	1.4	-	-	2.2e-14	1.2
rs3821236	2q32.3	*STAT1*	G>A	-	-	-	-	-	-	-	-	7e-05	1.1
rs7574865	2q32.3	*STAT4*	G>T	-	-	-	-	-	-	0.01	0.8	0.00038	0.9
rs6752770*	2q32.3	*STAT4*	A>G	-	-	-	-	-	-	-	-	8.1e-05	1.1
rs3087243	2q33.2	*CTLA4*	G>A	-	-	-	-	-	-	0.0011	1.2	2.3e-17	1.2
rs3828309	2q37.1	*ATG16L1*	G>A	-	-	-	-	-	-	-	-	-	-
rs12620999*	2q37.3	*COPS8*	T>C	-	-	-	-	-	-	-	-	-	-
rs11568821*	2q37.3	*PDCD1*	G>A	-	-	0.0035	0.77	-	-	-	-	-	-
rs6441961	3p21.31	*CCR2*	C>T	-	-	-	-	-	-	-	-	0.00028	0.91

				GADA		I-A2A		PCA		TPOA		T1D	
SNP	Chr	Closest Gene	Alleles	P	OR	P	OR	P	OR	P	OR	P	OR
rs730566*	3p21.31	*CCDC51*	C>A	-	-	-	-	-	-	-	-	-	-
rs9858542	3p21.31	*APEH*	G>A	-	-	-	-	-	-	-	-	-	-
rs3197999	3p21.31	*MST1*	G>A	-	-	-	-	-	-	-	-	-	-
rs6445975*	3p14.3	*PXK*	T>G	0.0088	1.2	-	-	-	-	-	-	-	-
rs17810546	3q25.33	*IL12A*	A>G	-	-	-	-	-	-	-	-	-	-
rs1464510	3q28	*LPP*	C>A	-	-	-	-	-	-	-	-	-	-
rs6853274*	4q24	*BANK1*	C>A	-	-	-	-	-	-	-	-	-	-
rs17388568	4q27	*ADAD1*	G>A	-	-	-	-	-	-	-	-	6e-06	1.1
rs2069762	4q27	*IL2*	A>C	-	-	-	-	-	-	0.0045	1.2	5.4e-07	0.89
rs6822844	4q27	*IL21*	G>T	-	-	-	-	-	-	-	-	-	-
rs2141258*	4q35.1	*CCDC111*	G>T	-	-	-	-	-	-	-	-	-	-
rs6897932	5p13.2	*IL7R*	C>T	-	-	-	-	-	-	-	-	0.0026	1.1
rs4613763	5p13.1	*SNORA63*	T>C	-	-	-	-	-	-	-	-	-	-
rs1050152	5q31.1	*SLC22A4*	C>T	-	-	-	-	-	-	-	-	-	-
rs6596075	5q31.1	*SLC22A5*	C>G	-	-	-	-	-	-	-	-	-	-
rs2188962	5q31.1	*AC116366.4*	C>T	-	-	-	-	-	-	-	-	-	-
rs10077785	5q31.1	*IRF1*	C>T	-	-	-	-	-	-	-	-	-	-
rs11747270	5q33.1	*ZNF300*	A>G	-	-	-	-	-	-	-	-	-	-
rs10036748*	5q33.1	*TNIP1*	C>T	-	-	-	-	-	-	-	-	-	-
rs6887695*	5q33.3	*IL12B*	G>C	-	-	-	-	-	-	0.005	1.1	-	-
rs6908425	6p22.3	*CDKAL1*	C>T	-	-	-	-	-	-	-	-	-	-
rs11755393*	6p21.31	*UHRF1BP1*	A>G	-	-	-	-	-	-	-	-	-	-
rs11755527	6q15	*BACH2*	C>G	-	-	-	-	-	-	9.7e-07	1.2	3.1e-08	1.1
rs6568431*	6q21	*PRDM1*	C>A	-	-	-	-	-	-	-	-	-	-
rs633724*	6q21	*ATG5*	C>T	-	-	-	-	-	-	-	-	-	-
rs6920220*	6q23.3	*TNFAIP3*	G>A	-	-	-	-	-	-	-	-	6.5e-05	1.1
rs1738074	6q25.3	*TAGAP*	C>T	-	-	-	-	-	-	-	-	0.00051	1.1
rs2301436	6q27	*FGFR1OP*	C>T	-	-	-	-	-	-	-	-	-	-
rs6959703*	7p21.3	*GLCCI1*	T>C	-	-	-	-	-	-	-	-	-	-
rs10156091	7p21.3	*ICA1*	C>T	-	-	-	-	-	-	-	-	-	-
rs1861525*	7p15.2	*CYCS*	A>G	-	-	-	-	-	-	-	-	-	-
rs10245867*	7p15.1	*JAZF1*	G>T	-	-	-	-	-	-	-	-	0.0011	1.1
rs921916*	7p12.2	*IKZF1*	T>C	-	-	-	-	-	-	-	-	-	-
rs10488630*	7q32.1	*IRF5*	A>G	-	-	-	-	-	-	-	-	-	-
rs11250113*	8p23.1	*XKR6*	C>T	-	-	-	-	-	-	-	-	-	-
rs13277113*	8p23.1	*BLK*	G>A	-	-	-	-	-	-	-	-	-	-
rs10903340*	8p23.1	*C8orf14*	T>G	-	-	-	-	-	-	-	-	-	-
rs868541*	8q12.1	*LYN*	C>T	-	-	-	-	-	-	-	-	-	-

SNP	Chr	Closest Gene	Alleles	GADA		I-A2A		PCA		TPOA		T1D	
				P	OR	P	OR	P	OR	P	OR	P	OR
rs1551398	8q24.13	*TRIB1*	A>G	-	-	-	-	-	-	-	-	-	-
rs10758669	9p24.1	*AL158147.17*	A>C	-	-	-	-	-	-	-	-	-	-
rs2812378	9p13.3	*CCL21*	A>G	-	-	-	-	-	-	-	-	-	-
rs6478108	9q32	*TNFSF15*	T>C	-	-	-	-	-	-	-	-	-	-
rs3761847	9q33.2	*TRAF1*	A>G	-	-	-	-	-	-	-	-	-	-
rs12722495	10p15.1	*IL2RA*	A>G	0.0066	0.73	-	-	-	-	-	-	1.4e-38	0.62
rs4750316	10p15.1	*PRKCQ*	G>C	-	-	-	-	-	-	-	-	-	-
rs11013210*	10p12.2	*ARMC3*	C>T	-	-	-	-	-	-	-	-	-	-
rs2666236	10p11.22	*NRP1*	G>A	-	-	-	-	-	-	-	-	1.3e-05	0.9
rs3936503	10p11.21	*CCNY*	G>A	-	-	-	-	-	-	-	-	-	-
rs1913517*	10q11.22	*LRRC18*	A>G	-	-	-	-	-	-	-	-	-	-
rs10995271	10q21.2	*ATQL4*	G>C	-	-	-	-	-	-	-	-	-	-
rs224136	10q21.2	*ATQL4*	C>T	-	-	-	-	-	-	-	-	-	-
rs11190140	10q24.2	*NKX2-3*	C>T	-	-	-	-	-	-	-	-	-	-
rs10886462	10q26.11	*GRK5*	A>G	-	-	-	-	-	-	-	-	-	-
rs4963128*	11p15.5	*PHRF1*	C>T	-	-	-	-	-	-	-	-	-	-
rs1004446	11p15.5	*IGF2AS*	A>G	-	-	-	-	-	-	-	-	2.5e-23	1.4
rs10836304*	11p13	*APIP*	A>G	-	-	-	-	-	-	-	-	-	-
rs7927894	11q13.5	*LRRC32*	C>T	-	-	-	-	-	-	-	-	-	-
rs503425*	11q23.3	*DDX6*	C>T	-	-	-	-	-	-	-	-	-	-
rs6590330*	11q24.3	*ETS1*	A>G	-	-	-	-	-	-	-	-	-	-
rs1805721*	12p13.31	*KLRG1*	G>A	-	-	-	-	-	-	-	-	0.0022	0.93
rs11175593	12q12	*LRRK2*	C>T	-	-	-	-	-	-	0.0087	2.8	-	-
rs1701704	12q13.2	*IKZF4*	T>G	-	-	-	-	-	-	-	-	4.5e-18	0.8
rs2292239	12q13.2	*ERBB3*	G>T	-	-	-	-	-	-	-	-	2.9e-27	1.3
rs1678542	12q13.3	*KIF5A*	C>G	-	-	-	-	-	-	-	-	0.00012	1.1
rs3184504*	12q24.12	*SH2B3*	C>T	0.0017	1.2	-	-	-	-	0.003	1.2	2e-38	1.3
rs428073*	12q24.23	*TAOK3*	T>C	-	-	-	-	-	-	-	-	-	-
rs662739*	12q24.31	*SPPL3*	C>T	-	-	-	-	-	-	0.00077	0.82	-	-
rs10847697*	12q24.32	*SLC15A4*	G>A	-	-	-	-	-	-	-	-	-	-
rs3764147	13q14.11	*C13orf31*	A>G	-	-	-	-	-	-	-	-	-	-
rs7333671*	13q22.1	*KLF12*	A>G	-	-	-	-	-	-	-	-	-	-
rs916977	15q13.1	*HERC2*	C>T	-	-	-	-	-	-	-	-	-	-
rs4778640*	15q25.1	*STARD5*	A>G	-	-	-	-	-	-	-	-	-	-
rs12708716*	16p13.13	*CLEC16A*	A>G	-	-	-	-	-	-	-	-	5e-14	1.2
rs4788084	16p11.2	*IL27*	C>T	-	-	0.0031	1.2	-	-	-	-	1.4e-08	0.87
rs9888739*	16p11.2	*ITGAM*	C>T	-	-	-	-	-	-	-	-	-	-
rs2076756	16q12.1	*CARD15*	A>G	-	-	-	-	-	-	-	-	-	-

				GADA		I-A2A		PCA		TPOA		T1D	
SNP	Chr	Closest Gene	Alleles	P	OR	P	OR	P	OR	P	OR	P	OR
rs991804	17q12	*CCL2*	C>T	-	-	-	-	-	-	-	-	-	-
rs2872507	17q12	*ORMDL3*	G>A	-	-	-	-	-	-	-	-	2.2e-06	1.1
rs7216389	17q12	*GSDMB*	C>T	-	-	-	-	-	-	-	-	4.1e-05	1.1
rs744166	17q21.2	*STAT3*	A>G	-	-	-	-	-	-	-	-	0.0023	0.93
rs2542151	18p11.21	*PTPN2*	T>G	-	-	-	-	-	-	-	-	3.8e-13	1.3
rs763361	18q22.2	*CD226*	C>T	-	-	-	-	-	-	-	-	1.3e-09	0.89
rs4807569	19p13.3	*SBNO2*	A>C	-	-	-	-	-	-	-	-	-	-
rs2304256*	19p13.2	*TYK2*	A>G	-	-	-	-	-	-	-	-	1.3e-10	-
rs2867437*	20q12	*PTPRT*	C>A	-	-	-	-	-	-	-	-	0.00061	1.1
rs1569723	20q13.12	*CD40*	A>C	-	-	-	-	-	-	-	-	-	-
rs4809330	20q13.33	*TNFRSF6B*	G>A	-	-	-	-	-	-	-	-	-	-
rs1736135	21q21.1	*CYCSP42*	T>C	-	-	-	-	-	-	-	-	-	-
rs2836878	21q22.2	*PSMG1*	G>A	-	-	-	-	-	-	-	-	-	-
rs3788013	21q22.3	*UBASH3A*	C>A	-	-	-	-	-	-	0.00099	1.2	1e-07	1.1
rs762421	21q22.3	*ICOSLG*	A>G	-	-	-	-	-	-	-	-	-	-
rs5754217*	22q11.21	*UBE2L3*	G>T	-	-	-	-	-	-	-	-	-	-
rs743777	22q12.3	*IL2RB*	A>G	-	-	-	-	-	-	-	-	1.7e-06	1.1
rs2071725*	22q13.2	*TTLL12*	G>A	-	-	-	-	-	-	-	-	-	-
rs4830808*	Xp22.2	*TLR8*	C>T	-	-	-	-	-	-	-	-	-	-
rs5979785*	Xp22.2	*TMSB4X*	T>C	-	-	-	-	-	-	-	-	0.00083	0.92
rs3027898*	Xq28	*IRAK1*	A>C	-	-	-	-	-	-	-	-	-	-
rs2071649*	Xq28	*OPN1LW*	A>C	-	-	-	-	-	-	-	-	-	-

Table S3: Genome-wide scan p-values and estimated minor allele odds ratios (OR) for GADA, IA-2A, PCA, TPOA and T1D associations for 135 SNPs associated with an autoimmune disorders [30,31] (excluding SNPs in the HLA locus or only T1D associated). When available, we used follow-up genotyping data in the maximum available sample size in the JDRF/WT T1D case control collection. The symbol * indicates that the SNP has been associated with systemic lupus erythematosus (see [31,32]). Only p-values and odds ratios more significant than 0.01 are shown (false discovery rate of 27%). The column Gene only refers to the nearest, or most likely candidate gene, and in most cases the causal gene may actually differ.
